# Reduction of Malaria Transmission to *Anopheles* Mosquitoes with a Six-Dose Regimen of Co-Artemether

**DOI:** 10.1371/journal.pmed.0020092

**Published:** 2005-04-26

**Authors:** Colin J Sutherland, Rosalynn Ord, Sam Dunyo, Musa Jawara, Christopher J Drakeley, Neal Alexander, Rosalind Coleman, Margaret Pinder, Gijs Walraven, Geoffrey A. T Targett

**Affiliations:** **1**Immunology Unit and Infectious Diseases Epidemiology Unit, Department of Infectious and Tropical DiseasesLondon School of Hygiene and Tropical MedicineUnited Kingdom; Liverpool School of Tropical MedicineUnited Kingdom

## Abstract

**Background:**

Resistance of malaria parasites to chloroquine (CQ) and sulphadoxine-pyrimethamine (SP) is increasing in prevalence in Africa. Combination therapy can both improve treatment and provide important public health benefits if it curbs the spread of parasites harbouring resistance genes. Thus, drug combinations must be identified which minimise gametocyte emergence in treated cases, and so prevent selective transmission of parasites resistant to any of the partner drugs.

**Methods and Findings:**

In a randomised controlled trial, 497 children with uncomplicated falciparum malaria were treated with CQ and SP (three doses and one dose respectively; *n* = 91), or six doses of artemether in fixed combination with lumefantrine (co-artemether [Coartem, Riamet]) (*n* = 406). Carriage rates of Plasmodium falciparum gametocytes and trophozoites were measured 7, 14, and 28 d after treatment. The infectiousness of venous blood from 29 children carrying P. falciparum gametocytes 7 d after treatment was tested by membrane-feeding of *Anopheles* mosquitoes.

Children treated with co-artemether were significantly less likely to carry gametocytes within the 4 weeks following treatment than those receiving CQ/SP (30 of 378 [7.94%] versus 42 of 86 [48.8%]; *p* < 0.0001). Carriers in the co-artemether group harboured gametocytes at significantly lower densities, for shorter periods (0.3 d versus 4.2 d; *p* < 0.0001) and were less infectious to mosquitoes at day 7 (*p* < 0.001) than carriers who had received CQ/SP.

**Conclusions:**

Co-artemether is highly effective at preventing post-treatment transmission of P. falciparum. Our results suggest that co-artemether has specific activity against immature sequestered gametocytes, and has the capacity to minimise transmission of drug-resistant parasites.

## Abstract 2

Do treatment regimens in malaria also affect transmission of parasites? In this African trial co-artemether worked significantly better than the chloroquine and sulphadoxine- pyrimethamine together

## Introduction

An increasing prevalence of drug-resistant parasites now threatens the efficacy of the antimalarial drugs chloroquine (CQ), sulphadoxine-pyrimethamine (SP), and amodiaquine (AQ) in sub-Saharan Africa [1,2,3]. In response, there is currently a concerted effort to evaluate new combinations of antimalarials [4]. In addition to direct therapeutic benefits, there is the important opportunity to select combination therapy that reduces post-treatment parasite transmission so as to counteract the selection and transmission advantage of drug-resistant parasites [4,5,6]. Recently published data show that the appearance in many geographical locations of parasites resistant to sulphadoxine and to pyrimethamine is not necessarily due to multiple de novo events, but rather that new resistant haplotypes have arisen extremely rarely and subsequently spread by selection pressure [7]. The goal of reducing this spread therefore requires that combination therapy minimises the number of treated individuals harbouring gametocytes, the transmissible sexual stages of the *Plasmodium* life cycle, and thus does not favour the survival and transmission of drug-resistant genotypes [8,9]. In a series of clinical trials in Farafenni, The Gambia, we have evaluated the effects of combination treatment on the subsequent infectiousness to mosquitoes of children treated for uncomplicated falciparum malaria. Children who received three daily doses of artesunate (AS) in addition to SP [6] or CQ [9,10,11] had significantly reduced gametocyte carriage and reduced infectiousness to mosquitoes compared to those who received either SP or CQ alone. However, the addition of three doses of artesunate did not wholly prevent gametocyte emergence in either case, and thus onward transmission occurred [6,9,10]. As SP-resistant parasites occur in The Gambia at low frequency [12], and CQ-resistant parasites are common [8], both SP/AS and CQ/AS will permit the transmission of resistant parasites from treated patients [9,10].

The co-formulation of artemether and lumefantrine, known as co-artemether (Coartem, Riamet), is an alternative artemisinin-based combination currently being evaluated in African clinical trials [13,14]. In areas of drug-resistant malaria, the World Health Organization (WHO) has recommended that co-artemether be used as a six-dose regimen, replacing the commonly used four-dose regimen [15,16]. Although no trials of the six-dose regimen have been reported in Africa, low gametocyte carriage rates have been observed in efficacy studies using the short treatment course [13,14]. There have been no studies of the effect of co-artemether treatment on transmission of parasites to the mosquito host.

A non-artemisinin combination under consideration in a number of African countries is CQ/SP, largely due to its low cost [17]. In The Gambia, where CQ has been front-line treatment policy up until 2004, clinical treatment failures with CQ monotherapy are approaching 20% in in vivo tests of drug efficacy [11]. It is hoped to extend the useful life of CQ by combining this drug with SP, which retains clinical efficacy of over 95% in The Gambia [12]. However, both of these drugs, when used alone, lead to a high prevalence of gametocyte carriage among treated patients [6,10]. Therefore, in contrast to artemisinin-based combinations, we are concerned that CQ/SP will not prevent transmission of CQ- and SP-resistant parasites from treated cases, resulting in a rapid deterioration in efficacy.

In this paper we present a study of Plasmodium falciparum transmission in Gambian children with uncomplicated malaria who were randomised to receive either six doses of co-artemether or CQ/SP. The major endpoints presented are gametocyte carriage rates, peripheral blood gametocyte density, and infectiousness to Anopheles gambiae mosquitoes of children carrying gametocytes 7 d after treatment.

## Methods

The study design comprised a single-blind, open-label, randomised, controlled trial in children with acute uncomplicated falciparum malaria, who were treated with either a combination of CQ and SP or six doses of co-artemether. Major endpoints were post-treatment gametocyte carriage and infectiousness to mosquitoes of children carrying gametocytes 7 d after treatment. The study protocol was approved by both the Joint Gambia Government/Medical Research Council Ethics Committee and the London School of Hygiene and Tropical Medicine Ethics Committee (Protocol S1) and is wholly compatible with the revised Helsinki Declaration of 1983 (Protocol S2; Table S1).

### Study Children and Treatment

Children 1–10 years of age attending Farafenni Health Centre, The Gambia, were screened at our recruiting clinic over a 12-wk period from September to December, 2002. Children with a temperature greater than 37.5 °C, or a history of fever and who were blood-film positive for P. falciparum at a density greater than 500 parasites per μl, and for whom signed informed consent was obtained, were eligible for recruitment into the study.

Exclusion criteria were: an inability to take drugs orally, treatment with antimalarial chemotherapy within the past 2 wk, carriage of circulating gametocytes at presentation, any evidence of chronic disease or acute infection other than malaria, domicile outside the study area (approximately 10-km radius), or any signs or symptoms of severe malaria. These latter included severe anaemia (peripheral blood packed cell volume [PCV] less than 20%), hyper-parasitaemia (more than 250,000 per μl of peripheral blood), respiratory distress (respiratory rate over 40 with two of the following: nasal flaring, intercostal indrawing, subcostal recession, or grunting), repeated generalized convulsions (three or more per 24 h or two witnessed seizures in 24 h), haemoglobinuria (dark red/black urine), jaundice, prostration, or circulatory collapse. Children were randomized to receive co-artemether or CQ/SP in the ratio 4.5:1 (see below).

Children in the CQ/SP group were given three daily oral doses of 10 mg of chloroquine base per kilogram of body weight, plus one half-tablet of SP (12.5 mg of pyrimethamine with 250 mg of sulphadoxine per half-tablet), and an additional one-quarter tablet for each 5 kg of body weight over 10 kg. Children in the co-artemether group received at each dose one half-tablet of co-artemether (20 mg of artemether with 120 mg of lumefantrine) per 5 kg body weight up to 24 kg (two tablets). Children 25 kg or heavier received the adult dose of three tablets. This treatment was given six times, at 0 h (i.e., in the clinic at approximately midday), 8 h (i.e., in the evening of the recruitment day), 20 h, 32 h, 44 h, and 56 h (± 90 min) after the initiation of treatment. This regimen is consistent with the manufacturer's recommendations.

Absorption of co-artemether is facilitated by fatty food, so the first dose was given to all children in both treatment groups with food (bread with a local oily sauce of beans) under the supervision of staff at the recruitment clinic. Each child was observed for 20 min after the first dose, and any child who vomited was administered a full replacement dose. All subsequent doses of both treatments were administered by a visiting field assistant at the child's home, accompanied by food provided by the child's family.

Each child also received 10 mg/kg paracetamol under supervision at recruitment, and parents or guardians were instructed to administer further doses every 6 h until the child's symptoms had subsided. Open-label chloroquine (Alkaloida), pyrimethamine-sulphadoxine (Pharmamed), and paracetamol (Echo International Services) were used. co-artemether was purchased from Novartis (Switzerland).

The treatment order was randomised by a Medical Research Council (MRC) statistician not engaged on the trial, such that two CQ/SP treatments occurred in each block of 11 assignments (see below). The treatment order, on slips of paper, was placed in identical envelopes numbered 1 to 550, each corresponding to a study subject number that was assigned to a single child by a study clinician prior to opening of the envelope. All staff engaged in the trial were blinded as to the treatment group of each child, apart from those in the recruiting clinic and the field assistants responsible for supervising administration of medication. Trial clinicians could not influence the choice of drug given. A physician at MRC Farafenni, who was not engaged in the study, fulfilled the role of trial monitor and held an additional copy of the randomised treatment code.

A major endpoint of the trial was infectiousness of patients to *Anopheles* mosquitoes, measured 7 d after treatment, so the study was designed so that approximately equal numbers of gametocyte carriers would be identified in both treatment groups at day 7 of follow-up. The treatment allocation ratio of 4.5:1 was based on an expected post-treatment day 7 gametocyte prevalence of 40%–50% among children receiving CQ/SP, as was observed in the previous year (Sutherland et al., unpublished data), and an estimate of day 7 gametocyte prevalence among co-artemether-treated children of between 5% and 10% [13,14]. The recruitment of 533 individuals was expected to provide at least 60 children divided equally between the two groups who were gametocytaemic on day 7 and prepared to donate venous blood for membrane-feeding, after gametocyte carriers were excluded at recruitment. These exclusions were necessary because children who present with peripheral gametocytaemia are very likely to carry gametocytes during the post-treatment period and to be infectious to mosquitoes, independent of the treatment regimen used [6,10,18]. This sample size would require dissection of approximately 1,200 mosquitoes. We have previously found that 5.3% of mosquitoes fed on blood donated by children treated with CQ/SP and carrying gametocytes at day 7 became infected, and that 20% of gametocyte carriers were infectious (Sutherland et al., unpublished data). This study was designed to provide a power of 90% at the 95% confidence level to detect a 3.5-fold reduction in the proportion of mosquitoes infected by gametocyte carriers on day 7.

Finger-prick peripheral blood from all children attending the recruitment clinic was obtained for thick blood-film examination, PCV determination, and, in the case of recruits, collection of a filter paper sample for subsequent nucleic acid extraction. Parasitological admission criteria were checked at the recruitment clinic using thick blood films stained with rapid Field's stain; 25 high-power fields were examined. Duplicate thick blood films from all recruits were returned to the laboratory, allowed to dry for 24 h, and stained with Giemsa. Two reads of 100 high-power fields were performed for asexual and sexual stage parasites. Parasitaemia was calculated as described [19].

Transport was provided to bring children to the MRC Field Station at Farafenni 7 d after treatment to be screened for circulating gametocytes as previously described [10]. Finger-prick samples for thick-blood film and haematocrit were obtained, and a field assistant fluent in four local languages posed a fixed list of questions to each guardian and/or child to determine if clinical signs or symptoms had resolved and if there had been any of a range of adverse events. Answers were recorded on a specific report form designed for this purpose. Thick-blood films were stained with Field's stain, and 100 fields were examined immediately for the presence of gametocytes. Guardians of any gametocyte carriers were asked if approximately 3 ml of venous blood could be taken from the child's arm for membrane-feeding of mosquitoes. Day 7 follow-up and gametocyte screening were performed on day 6 in the case of eight individuals for whom day 7 fell on a religious festival. Children who were not available to be brought to the MRC laboratory on the morning of day 7 were followed up at home by a field assistant whenever possible, and the same fixed list of questions posed. However, such children were not able to be venous blood donors for mosquito feeding. Field assistants made further visits to each child on post-treatment days 14 and 28 to obtain finger-prick peripheral blood for thick films and dried filter-paper blood spots for parasite DNA extraction. At each contact with study participants, field staff asked the patient and/or guardians whether any symptoms indicative of clinical malaria had persisted or returned since treatment, and whether the child had experienced any other adverse events. This information was recorded on a follow-up form. Participants were encouraged to attend the recruiting clinic at any time should the child become sick.

### Estimating Duration of Gametocyte Carriage

Gametocyte carriage was estimated by scoring each individual as gametocyte positive or not on each of the follow-up days (0, 7, 14, or 28) [20]. Given the reported half-life of gametocytes of 2.5 d [21], a single gametocyte-positive slide was assumed to represent gametocytes circulating in the peripheral blood for 2.5 d. When patients had blood films that were gametocyte positive in successive weeks, the estimated time of gametocyte carriage was the interval between observations plus 2.5 d. It was assumed that a single negative or missing slide between two positive slides had a greater than 50% chance of being positive [22]. Between-group comparison of mean duration of gametocyte carriage was by the rank-sum test of Wilcoxon.

### Membrane Feeding and Mosquito Dissection

Venous blood samples were obtained from children who were gametocytaemic (limit of detection, 5 gametocytes/μl) on day 7, who had a PCV greater than 20%, and whose parent or guardian gave specific consent for the procedure. If the number of gametocyte-positive patients was greater than the number of cages of mosquitoes available for feeding on a particular day, consenting gametocyte carriers were selected in the order in which they were finger-pricked, which was not dependent on study identification numbers or treatment group. The mosquito feeds were performed as described elsewhere [6,10]. In brief, venous blood in citrate-phosphate dextrose (anticoagulant) was centrifuged and the plasma removed. After being washed, the red blood cell pellet was resuspended to a PCV of 33% in pooled type AB serum from European donors with no history of malaria exposure. This allowed us to reduce the possible effects of residual drug or of antibody in the blood meal [10], although some CQ may be carried over within erythrocytes. Each suspension then was fed to approximately 50 5-d-old female *A. gambiae s.s.* mosquitoes via an artificial membrane attached to a water-jacketed glass feeder maintained at 37 °C. Mosquitoes were from an established colony maintained by feeding on rabbits. Mosquito mid-guts were dissected 7–8 d later, and the number of malaria oocysts on the mid-gut recorded.

### Molecular Genotyping

P. falciparum genotypes circulating in children with detectable asexual parasitaemia during post-treatment follow-up were compared with those present in the same child prior to treatment. DNA was extracted from dried blood spots using a Chelex-based method [8]. Alleles of the polymorphic locus *pfmsp2* were compared between pre- and post-treatment parasite isolates by PCR [8,23]. The procedure of Cattamanchi et al. [24] was followed, in that indeterminate samples in which a majority of novel bands appeared in the post-treatment infection were scored as new infections. There were only seven such indeterminate infections among the 44 successfully typed.

### Data Analysis

Data were analysed using Stata, version 8 (StataCorp, Texas). Proportions were compared using the chi-square statistic or Fisher's exact test. In the case of mosquito dissection data, the confidence intervals (CIs) of proportions were estimated by bootstrapping to take account of clustering within feed-cages. Normally distributed continuous variables were compared using the t-test of Student.

Gametocyte density was compared between groups using the ratio of arithmetic means, as in a previous study [10]. This was done by fitting generalized linear models with a logarithmic link function (the “nbreg” command in Stata), i.e., modelling the logarithm of the (arithmetic) mean gametocyte density [25]. Ratios of mean densities were obtained by exponentiating the relevant regression coefficients. We favour this approach because the test statistic (mean ratio) is intuitively simple, the negative binomial function gives a good fit to the typically skewed distributions observed, and because all data points (including zeroes) are included. Thus differences in gametocyte prevalence, as well as density, contribute to the mean ratio.

## Results

Of 3,167 febrile children, 1,328 were infected with microscopically-confirmed P. falciparum and did not have circulating gametocytes. Eighty-three children (6.3%) harboured both gametocytes and trophozoites, and were not recruited. A total of 497 eligible children were enrolled in the trial and randomised between the treatment groups. This was a smaller number than predicted, because of lower rainfall than in previous seasons, and thus reduced malaria transmission. The reason for ineligibility was recorded for 82% of non-recruits. These were: residence outside the study area (26.1%), parasitaemia below the threshold (16.5%), consent withheld (15.9%), anaemia (PCV below 20%) (7.6%), participation in another research study (4.4%), recent antimalarial use (3.8%), hyperparasitaemia (2.9%), other signs of severe malaria (1.6%), vomiting (0.7%), other disease present (0.6%) and other reasons not included in the above (19.9%).

**Figure 1:**
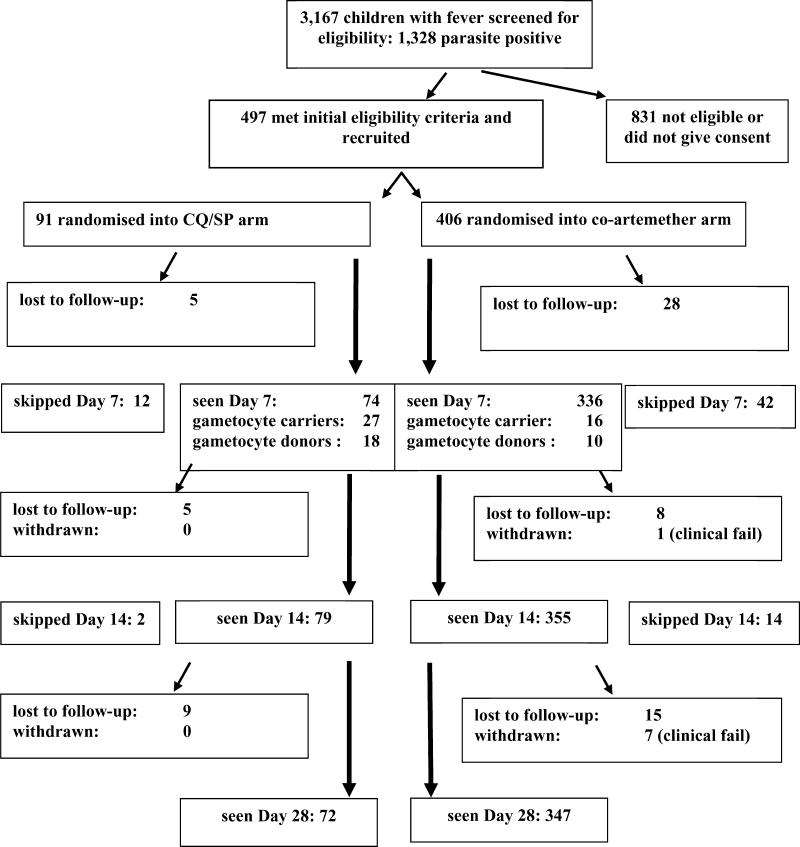
Profile of the Study Ten and 38 children in the CQ/SP and co-artemether groups, respectively, who were not seen on day 7 were seen on day 14. Two and four children, respectively, who were not seen on day 7 or day 14 were seen on day 28. Ten children in the co-artemether group not seen on day 14 were seen on day 28.

Ninety-one children received CQ/SP and 406 received co-artemether. Baseline characteristics of these 497 enrolments are presented in Table 1. Two children enrolled and treated in the CQ/SP group, and six in the co-artemether group, were found to have zero parasitaemia upon subsequent examination of Giemsa-stained duplicate slides. Similarly, four (4.4%) of the CQ/SP group and 26 (6.5%) of the co-artemether group were found to have been carrying peripheral gametocytes at presentation that had not been identified in the recruitment clinic. These and all other subjects randomised between treatments were retained in the primary analysis under our intention-to-treat protocol. Eighty-eight (96.7%) of the CQ/SP group, and 379 (93.4%) of the co-artemether group, were visited five times by a field assistant in the 56 h following recruitment, and received the full regimen allotted under supervision. Seventy-two (79.1%) and 347 (85.5%) of the CQ/SP and co-artemether groups, respectively, completed follow-up to day 28, although 12 and 46 of these, respectively, skipped day 7, day 14, or both. A profile of the study is given in Figure 1.

**Table 1:**
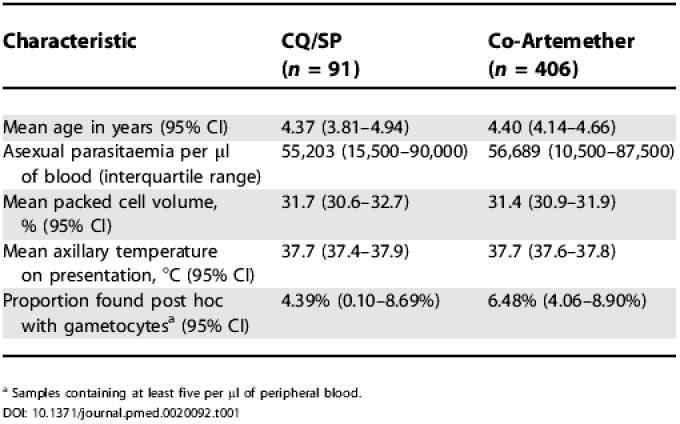
Baseline Characteristics of Enrolled Patients

^a^ Samples containing at least five per μl of peripheral blood

### Adverse Advents

No serious adverse events occurred in either treatment group, and there were no deaths among children recruited into the study. Minor complaints noted at the day 7 laboratory visit included headache (12% and 11% in the CQ/SP and co-artemether groups, respectively), anorexia (12% and 16%), diarrhoea (7% and 4%), abdominal pain (5% and 5%), and pruritis (1% and 1%).

### Gametocyte Prevalence and Density

**Figure 2:**
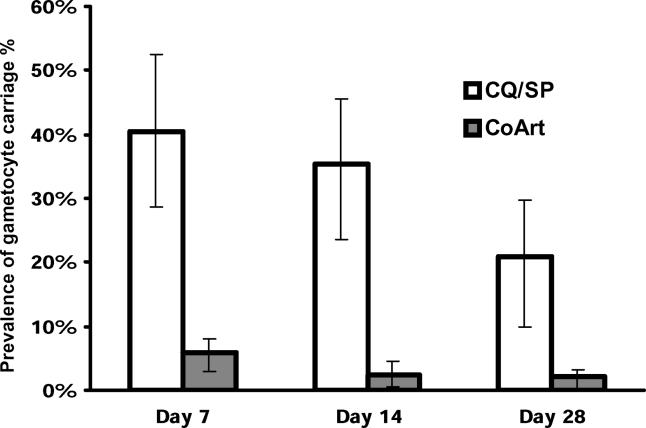
Gametocyte Carriage after Treatment with CQ/SP or Co-Artemether Point-prevalence of gametocyte-positive thick films in both treatment groups at 7, 14, and 28 d post-treatment (limit of detection: five gametocytes per μl of peripheral blood). Error bars represent 95% CI calculated from the binomial distribution. Denominators for CQ/SP and co-artemether (CoArt) groups are, respectively, 74 and 336 (day 7), 79 and 356 (day 14), and 72 and 355 (day 28).

Children treated with co-artemether were significantly less likely to carry gametocytes during follow-up than children treated with CQ/SP (Figure 2; *p* < 0.001 at each of days 7, 14, and 28). In the CQ/SP group, 42 of 86 (48.8%) evaluable children had detectable peripheral gametocytaemia at some point over 28 d of post-treatment follow-up, compared to 30 of 378 (7.9%) evaluable children in the co-artemether group (relative risk 6.15; 95% CI, 4.10–9.23; *p* < 0.001). The mean duration of gametocyte carriage was also significantly lower in the co-artemether group (0.3 d compared to 4.2 d in the CQ/SP group; range in both groups 2.5–23.5 d; *p* < 0.0001).

The ratio of mean peripheral gametocyte density in all children at each follow-up visit is presented in Table 2. Six of the eight children in the co-artemether group who were gametocyte-positive at day 28 also harboured trophozoites, suggesting a new infection emerging from the liver, whereas only one of 15 gametocyte carriers in the CQ/SP group also harboured trophozoites at day 28 (odds ratio = 42; 95% CI, 2.4–2,061; Fisher's exact *p* = 0.002).

**Table 2:**
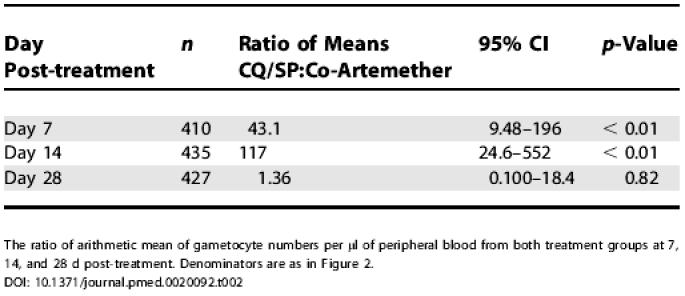
Ratio of Means of Gametocyte Density in Peripheral Blood after Treatment with CQ/SP or Co-Artemether

The ratio of arithmetic mean of gametocyte numbers per μl of peripheral blood from both treatment groups at 7, 14, and 28 d post-treatment. Denominators are as in Figure 2

### Infectiousness of Treated Children

399 children were seen at the MRC laboratories on day 7, 73 in the CQ/SP group and 326 in the co-artemether group. There were 27 children (37.0%) in the CQ/SP group and 16 children (4.9%) in the co-artemether group identified as gametocyte carriers by our rapid screening procedure (*p* < 0.001). Subsequent definitive microscopy with the Giemsa-stained duplicate thick films gave good concordance with the screening result (positive predictive value 90.2%; specificity 98.8%). Explicit consent for venous blood donation for membrane-feeding of mosquitoes was obtained from 29 (67.4%) of these 43 children: 19 (67.9%) of the gametocyte positives in the CQ/SP group and 10 (62.5%) of those in the co-artemether group. Mean ages among these 29 children were 3.3 years (95% CI, 2.4–4.3) and 4.2 years (2.7–5.7) in the two treatment groups, respectively.

Results of the transmission experiments are given in Table 3. Whereas seven children (36.9%) among the CQ/SP gametocyte donors were infectious to mosquitoes, none of the gametocyte donors in the co-artemether group was infectious. The infectious proportion observed in the CQ/SP group was higher than expected from an earlier study in which 20% of CQ/SP treated children were infectious, perhaps due to the use here of an established blood-fed mosquito colony, which leads to better insect survival. In the current study, we dissected an average of 18.6 mosquitoes (95% CI, 16.8–20.4) per infection experiment, compared to 14.8 mosquitoes (95% CI, 12.5–17.1; two-tailed *p* = 0.021) in a similar study performed in 2001 that used F1 progeny of wild-caught female *Anopheles*. The data in Table 3 show that gametocyte donors in the co-artemether group carried fewer gametocytes, and were as a result significantly less infectious than those in the CQ/SP group, consistent with previous studies [6,10]

**Table 3:**
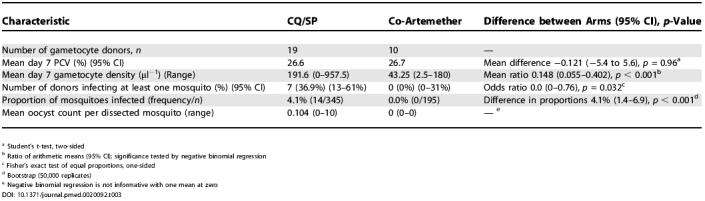
Profile of 29 Children Who Donated Gametocyte-Positive Blood at Day 7 and Results of Transmission Experiments

^a^ Student's t-test, two-sided

^b^ Ratio of arithmetic means (95% CI); significance tested by negative binomial regression

^c^ Fisher's exact test of equal proportions, one-sided

^d^ Bootstrap (50,000 replicates)

^e^ Negative binomial regression is not informative with one mean at zero

### Clinical and Parasitological Efficacy of CQ/SP and Co-Artemether

**Figure 3:**
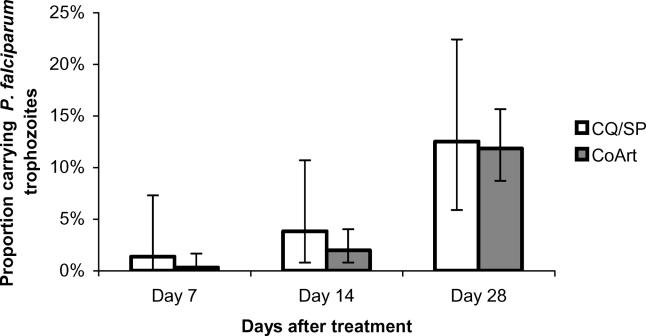
Crude Parasitological Failure after Treatment with CQ/SP or Co-Artemether Point-prevalence of trophozoite-positive thick films in both treatment groups at 7, 14, and 28 d post-treatment (limit of detection: five trophozoites per μl of peripheral blood). Error bars represent 95% CI calculated from the binomial distribution. Denominators for CQ/SP and co-artemether (CoArt) groups are, respectively, 74 and 336 (day 7), 79 and 356 (day 14), and 72 and 355 (day 28).

Among eligible children both regimens employed were highly efficacious against acute uncomplicated falciparum malaria. Clinical treatment failure, defined as the persistence or recurrence of malaria symptoms with detectable peripheral trophozoites, occurred in 8 cases (1.6%), all of which were in the co-artemether treatment group. One of these patients received only a single observed dose of co-artemether at the recruiting clinic, the other seven cases received the full six-dose regimen under supervision. All eight clinical failures occurred 2 wk or more after treatment. The cumulative 28-d clinical failure rate among children under 5 years of age was 2.55%, and among those 5 years and over 0.45%; (relative risk 5.65; 95% CI, 0.70–45.6; one-sided Fisher's exact *p* = 0.064). Parasitological treatment failure, defined as carriage of asexual parasites (trophozoites) during post-treatment follow-up, occurred in fewer than 5% of patients in both treatment groups up to day 14, but rose to approximately 12% in both groups by day 28 (Figure 3).

Interpretable PCR data were obtained for 44 of the 60 post-treatment asexual parasite infections identified by microscopy. Parasites harboured at the time of treatment failure were indistinguishable from pre-treatment parasites by *msp2* PCR genotyping in 15 of the 44 cases for which the data were interpretable. Post-treatment parasites in five of the nine children in the CQ/SP group were caused by recrudescent infections compared to ten of 35 in the co-artemether group. After correction of the 28-d cumulative parasitological failure rate using these ratios, it was estimated that co-artemether was 96.1% efficacious over 28 d, resulting in a total of 14 recrudescent infections, compared to 91.1% for CQ/SP, resulting in seven recrudescent infections (odds ratio = 0.413; 95% CI, 0.150–1.26; *p* = 0.058).

## Discussion

Co-artemether, administered to children with uncomplicated falciparum malaria as a six-dose regimen, is highly effective at reducing the prevalence and duration of gametocyte carriage, the density of peripheral gametocytaemia among carriers, and the infectiousness to mosquitoes of carriers at day 7 compared to a combination of CQ and SP. In the co-artemether group, none of the ten children who carried gametocytes at day 7 and donated blood for membrane-feeding of mosquitoes was found to be infectious. In contrast, 7 (36.9%) of the 19 gametocyte donors in the CQ/SP group were infectious, largely due to a significantly higher gametocyte density than in the co-artemether group. This suggests that co-artemether has a specific action against developing gametocytes, as any gametocytes that appeared on or before day 7 must have been committed to sexual differentiation at the time of treatment, because of the period of 8–10 d of sequestered sexual development that occurs before gametocytes are detectable in the periphery [26]. This anti-gametocyte activity is additional to the anti-trophozoite and anti-schizont effects of co-artemether, which contribute to the very low gametocyte carriage rates observed later in follow-up. Importantly, we also found evidence that post-treatment carriage of both trophozoites and gametocytes in the co-artemether group, but not the CQ/SP group, was due mainly to new emergent infections, rather than recrudescent infections, which suggests that artemether-lumefantrine reduces transmission of recrudescent, resistant parasites compared to CQ/SP. Genotyping of these infections at resistance-associated loci is underway to test this further.

The potential of artemisinin-containing combination therapies to reduce gametocyte carriage rates, and therefore transmission of falciparum malaria, was first shown by Price et al. [5]. This study, among refugee camps on the Thailand-Burma border, showed that deploying the combination of mefloquine with artesunate significantly reduced the incidence of malaria cases over a few years in this low-transmission area where asymptomatic infections are rare and thus drug pressure is very high. We have extended this work to a setting in sub-Saharan Africa where transmission is high and seasonal, asymptomatic infections are common, and as few as 20% of slide-positive infections are treated [27]. Transmission reduction at the population level through the treatment of clinical cases with artemisinin-containing combination therapy is unlikely in this setting, and so our strategy has been to use membrane-feeding of Anopheles gambiae mosquitoes to measure the infectiousness of individual treated children who become gametocyte carriers after treatment [6,10]. Those children who are carrying gametocytes prior to treatment are likely to become infectious regardless of the treatment received. We can estimate the proportion of treated children estimated to be infectious by multiplying the proportion of gametocyte donors that were infectious by the prevalence of gametocyte carriage at day 7. Using the data in Table 3, % (95% CI, 0–1.9% ) of children treated with six doses of co-artemether will be infectious to mosquitoes at day 7. For CQ/SP, the corresponding estimate is 15.8% (5.6%–26.0%). We have previously estimated that the mean infectious proportion in control serum membrane-feeding experiments for SP plus artesunate (three doses) in 1999 was 4%, and for CQ plus artesunate (three doses) was also 4% [6,10].

Six doses of co-artemether produced the lowest gametocyte carriage rates we have seen in 5 years of consecutive transmission studies in Farafenni [6,10], excluding patients harbouring gametocytes at day 0. Although 2002 had lower rainfall than any of the previous 10 years, there were nevertheless significant numbers of gametocyte carriers, many of them infectious, in the CQ/SP group in this study. From a public health point of view, the effect of each regimen on all children presenting for treatment, including gametocyte carriers, is of interest. In co-artemether-treated children who had unidentified circulating gametocytes at recruitment, seven of 20 (29.2%) carried gametocytes at 7 d, compared to 13 of 312 (4.2%) of those without circulating gametocytes at the time of treatment (Fisher's exact test, *p* < 0.01). This demonstrates that children presenting with gametocytaemia at day 0 make a disproportionate contribution to day 7 gametocytaemia. Inclusion, hypothetically, of the 83 non-recruited gametocyte carriers in addition to the 30 that were, predicts that only 8% of children in the co-artemether group would have harboured day 7 gametocytes, compared to 45% in the CQ/SP group. Co-artemether is thus likely to be highly effective at reducing post-treatment gametocyte carriage among children irrespective of gametocytaemia at presentation.

At day 28, gametocyte carriage in the co-artemether group was associated with evidence of a new infection recently emerged from the liver. This was not the case with CQ/SP. Therefore, at day 28 a significant proportion of children treated with CQ/SP will harbour gametocytes that originated from the treated clinical infection. These gametocytes and their asexual progenitors have been under sustained drug selection, given the elimination half-lives of CQ, des-ethylchloroquine (an active metabolite of CQ), sulphadoxine and pyrimethamine: 5.9 d, 8.5 d, 10.9 d, and 2.9 d, respectively when given simultaneously as CQ/SP [28]. CQ continues to be gradually released back into the circulation from host tissues for at least 30 d [29]. In contrast, artemether and lumefantrine are cleared from the body with terminal elimination half-lives of 0.8 h and 4.5 d, respectively, after administration of co-artemether [30]. Thus, parasites detected in co-artemether-treated children during follow-up have, on average, experienced a shorter period of selective drug pressure than those in CQ/SP-treated children. Nevertheless, lumefantrine is “unprotected” for several days following the elimination of artemether and its metabolites, and so we cannot rule out the emergence of resistance to lumefantrine under widespread use of co-artemether.

We found that co-artemether is better than CQ/SP at preventing recrudescence of treated parasites. However, as seen with artemisinins in other areas of moderate to high malaria transmission, co-artemether has the disadvantage of permitting rapid re-infection, which can lead to new clinical attacks. This is due to absence of the prophylactic effect afforded by a pharmacological “tail,” such as that seen with both SP and CQ. From our co-artemether data, the 28 d new infection rate in our study population is approximately 10% with fewer than a fifth of these experiencing a new clinical attack. Although this reinfection/clinical infection rate may be even higher in other settings, we believe it would be offset by the ability of co-artemether to reduce selection and transmission of drug resistant parasites, and thus make possible sustainable improvements in both therapeutic and public health outcomes. In contrast, as our data show, the pharmacological persistence of CQ and SP eliminates sensitive parasites emerging from the liver, and so permits a much smaller proportion of new infections (3.5%). We predict that CQ/SP thus selects for any resistant mutants present in the starting population, leading to increased onward transmission of resistant parasites, and reduced onward transmission of sensitive parasites.

There are difficulties with the introduction of co-artemether as a first-line treatment for malaria in Africa. Firstly, as the six-dose regimen is the most likely to provide the treatment and transmission-reducing benefits needed, there may be problems with treatment compliance. Secondly, the requirement of oily food for adequate absorption may lead to inadequate drug levels in the blood of many treated individuals. Effectiveness studies that examine these indicators under conditions as close as possible to the normal clinical setting, and with both therapeutic and transmission endpoints, are thus of great importance. Nevertheless, the therapeutic success of co-artemether as a front-line treatment policy has been demonstrated in KwaZulu–Natal, South Africa, where it replaced SP [31]. The impact of this policy on post-treatment transmission in this region is yet to be determined.

We have shown that, compared to CQ/SP, co-artemether significantly reduces post-treatment gametocyte carriage, peripheral gametocyte density and infectiousness of gametocyte carriers. There is little recrudescence of parasites under co-artemether, and the majority of gametocyte carriers are associated with new infections that will have been subject to little or no drug pressure. We are not suggesting that introduction of co-artemether as first-line treatment policy in The Gambia or elsewhere is likely to reduce overall transmission of P. falciparum within the community, but rather that selective transmission of resistant parasites in treated patients would be greatly reduced. The benefit is because drug pressure is exerted only on these treated infections. Therefore, the use of co-artemether is likely to have measurable public health benefits in reducing the impact of drug resistance. We predict that these benefits would prove far more robust than those transiently enjoyed after the initial deployment of SP in Africa.

## Supporting Information

### Trial Registration

The Wellcome Trust Project number is 061910. An International Standard Randomised Controlled Trial Number (ISRCTN) is being applied for.

Protocol S1 Ethics Permission (283 KB DOC).

Protocol S2 Study Protocol (99 KB DOC).

Table S1 Consort Table (55 KB DOC).

**Patient Summary**

**Background**

Treatment studies of malaria often focus on the whether parasites have become resistant to anti-malarial treatment. However, an ideal treatment would be one that not only cured the patient but also prevented further transmission of the parasite, especially those that carry resistance, to mosquitoes and hence to other individuals.

**What Did the Researchers Do?**

They randomised 497 children with uncomplicated falciparum malaria (the most serious kind) in Gambia to either a combination of two drugs (chloroquine and sulphadoxine-pyrimethamine [CQ/SP]) to which many malarial parasites have become resistant, or a new drug, artemether, in fixed combination with lumefantrine (co-artemether, brand names Coartem and Riamet). Within the 4 weeks following treatment they found that patients treated with co-artemether were much less likely to contain the stage of the malarial parasite that is infectious, that they carried the parasites for a shorter time, and that blood from the children treated with co-artemether was less able to infect mosquitoes.

**What Do These Findings Mean?**

The new combination substantially reduces the chances of malaria being transmitted from infected patients, and this finding should be taken into account when deciding which drugs are the preferred choice for treatment.

**Where Can I Get More Information?**

The Wellcome Trust has information on malaria: http://www.wellcome.ac.uk/en/malaria/

The World Health Organisation has a programme called Roll Back Malaria, which has various initiatives: http://www.who.int/topics/malaria/en/

The Gates foundation funds research on malaria, one arm of which is the Gates Malaria Partnership: http://www.lshtm.ac.uk/gmp/

## Abbreviations

AQ: amodiaquine
AS: artesunate
CQ: chloroquine
MRC: Medical Research Council
PCV: packed cell volume
SP: sulphadoxine-pyrimethamine
