# Supplementary figures and images for: Reduction of Malaria Transmission to Anopheles Mosquitoes with a Six-Dose Regimen of Co-Artemether
Source: PLoS Med. 2005 Apr 26;2(4):e92. doi: 10.1371/journal.pmed.0020092 (PMC1087200; doi:10.1371/journal.pmed.0020092)

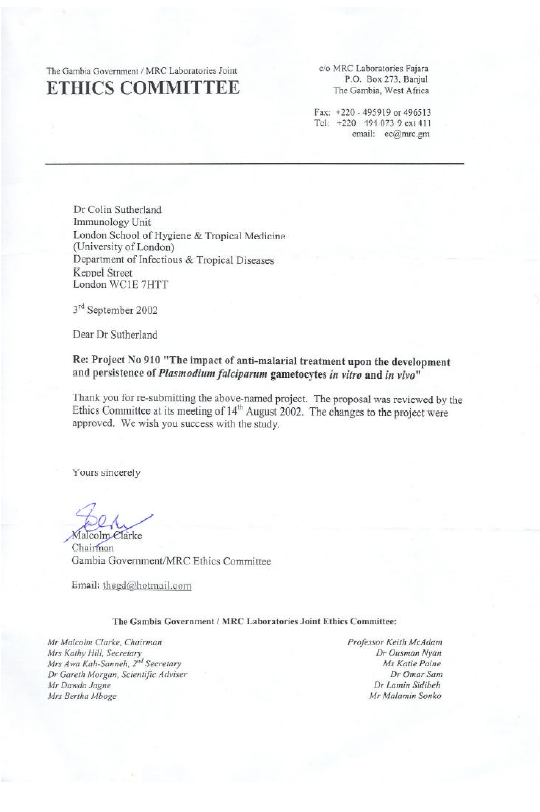

Supplement: Protocol S1 — (283 KB DOC). [file pmed.0020092.sd001.doc]
