# Supplementary material for: Reduction of Malaria Transmission to Anopheles Mosquitoes with a Six-Dose Regimen of Co-Artemether
Source: PLoS Med. 2005 Apr 26;2(4):e92. doi: 10.1371/journal.pmed.0020092 (PMC1087200; doi:10.1371/journal.pmed.0020092)
Supplement: Protocol S2 — (99 KB DOC). [file pmed.0020092.sd002.doc]

# Medical Research Council Laboratories, Fajara. Application to undertake a research project.

**NB: This document is as submitted in 2002, although the budget has been removed, the consent form appended, and the exclusion of gametocyte carriers added (p9). This was decided prior to commencement of recruiting in 2002, and is explained in the manuscript. This exclusion was implicit in the original sample size calculation in this protocol (p8).**

**__________________________________________________________________________**

#### A Summary Information

**A1 Title of project**

The impact of anti-malarial treatment upon the development and persistence of *Plasmodium falciparum* gametocytes *in vitro* an*d in vivo.*

**A2 Investigators (Principal Investigator first)**

| **Name** | Institution | Position |
| --- | --- | --- |
| Sutherland, Colin J. | London School of Hygiene and Tropical Medicine Medicine | Lecturer |
| Targett, Geoffrey A.T. | LSHTM | Prof. of Parasite Immunology |
| Dunyo, Sam | MRC Field Station, Farafenni | Clinical Epidemiologist |
| Walraven, Gijs | MRC Field Station, Farafenni | Field Station Head |
| Juwara, Musa | MRC Field Station, Farafenni | Entomologist |
| Chibuzo Nwakanma | MRC Field Station, Farafenni | Molecular Biologist |
| Milligan, Paul | MRC Laboratories, Fajara | Statistician |
| Pinder, Margaret | MRC Laboratories, Fajara | Programme Head |
| Camara, Bekai | AFPRC Hospital, Farafenni | CEO |
| Sillah, Jackson | WHO, The Gambia | RBM Co-ordinator |

**Who will introduce the proposal at SCC?**

Dr Margaret Pinder

**A3 Location(s) of research**

Please list all the places where the research will take place including field sites or health facilities

MRC Field Station, Farafenni

Farafenni AFPRC Hospital/MCH clinic

London School of Hygiene and Tropical Medicine

**A4 Proposed start date and duration in months**

In giving a date please bear in mind the timescale for decision-making by SCC, the Ethical Committee and any other institutions whose agreement is needed, and the time needed to organise the resources required.

September 2002, for 3.5 months at Farafenni; data analysis to follow in London and The Gambia.

NB. This study follows those of 2000 and 2001 by the same investigators, in which we measured post-treatment transmission among children treated with several different antimalarial regimens.

**A5 Reference (office use)**

**Scientific Co-ordinating Committee No 910**

**Ethical Committee No 910**

**A6 Summary of project, long term objectives and specific aims (not more than 200 words)**

This section is very helpful to the Committees in determining quickly the main features of the study, and should be as clear and concise as possible. It should cover the key objectives and endpoints and, if the project is hypothesis driven, then the hypothesis should be stated here.

Widespread resistance of *Plasmodium falciparum* to commonly-used drugs has lead to the formulation and testing of new combinations of anti-malarials, particularly those including one of the artemisinins. This proposal is one in a series of trials with the long-term objective of identifying highly efficacious drug combinations that also reduce or prevent post-treatment transmission of *P. falciparum*. Our studies in 1998-2001 have shown that:

- Sulphadoxine/pyrimethamine (SP; Fansidar®) treatment is followed by high gametocytaemia in a large proportion of subjects.
- Compared to SP, artesunate (AS) + SP reduces, but does not prevent, transmission of *P. falciparum* to mosquitoes (9).
- the prevalence of resistance to chloroquine (CQ) is sufficiently high in the Farafenni area to severely compromise CQ + AS as a treatment for uncomplicated malaria.
- CQ + AS reduces transmission at day 7, but this benefit is probably transient, as gametocyte carriage increases significantly in the latter half of follow-up, and these gametocytes are likely to have CQ-resistant genotypes.

Thus whereas both of the AS combinations we have tested do reduce the infectiousness of treated children, neither can completely prevent transmission. The CQ + AS combination has the added disadvantage of poor efficacy, whereas SP + AS is efficacious but permissive of post-treatment transmission. The results of this series of trials rank SP + AS as the best combination tested to date, suggesting that the anti-folate class of drugs make good partners for AS. The chief disadvantages of this regimen are that transmission can occur in 5 to 10 % of infected children treated with this combination, and that SP has proved to be very vulnerable to rapidly progressing drug resistance in other African settings. This is partially due to its very long half-life in the patient’s circulation (2). It follows that any parasite remnant that survives AS (a drug that is rapidly cleared) will be under a lengthy period of selection by SP. The prevalence of resistance to SP is low around Farafenni, but greater than zero (11) so that widespread administration of this combination may lead to the emergence of SP resistance as a serious threat to long-term efficacy.

We have not met our chief aim of identifying antimalarial combinations that are safe, affordable, highly efficacious and which reduce or completely ablate post-treatment transmission when used to treat Gambian children with uncomplicated falciparum malaria. A new candidate for transmission-reducing treatment is required. We propose to test the infectiousness of children treated with artemether + benflumatol (Co-Artemether), as clinical trials in The Gambia have shown this drug to be highly efficacious, and suggest that post-treatment gametocyte-carriage is low (12, 13). Gametocyte prevalence and density, and the infectiousness to mosquitoes of treated persons have not been systematically measured for Co-Artemether.

A7 Checklist/Signatures

**Please complete the following checklist and comment as appropriate.** This section is designed to ensure that all the planning steps have been taken that are needed for successful project. For projects at the MRC Laboratories, Programme Heads will help visiting workers, and others preparing proposals at a distance, to ensure liaison with key individuals who need to be consulted locally.

1. **Has the project been discussed and cleared with the institutions in which research will be carried out including health services to which the study will need access?**

Yes

1. **Have all investigators and collaborators given their agreement to take part in the study as described?**

Yes

1. **Has a CV been attached for the principal investigator if unknown to the Committee?**

Not applicable

1. **Have ethical issues been addressed? Give details in section C.**

Yes

1. **Have safety issues been addressed? Please give details**

Yes

1. **Will the project require data and/or materials to be taken out of The Gambia? If so please give details** Data will be analysed at LSHTM in collaboration with MRC staff in The Gambia. We plan to take the following material out of The Gambia to either LSHTM or the University of Edinburgh (*):

- filter-paper blood spots for parasite genotyping studies
- small (~50μl) blood samples for parasite RNA extraction *
- alcohol-fixed oocysts from mosquito guts *
- frozen sera used in transmission experiments
- paraformaldehyde-fixed parasites on microscope slides for immunofluoresence*

1. **For projects to be carried out at MRC Laboratories: Have the following been consulted about the support services, resources and working space required?**

|  | **Consulted: Yes/No/Comment** |
| --- | --- |
| Laboratory Manager (including safety issues) | Ongoing |
| Director of Clinical Services | Project at AFPRC hospital/MCH clinic, discussed with CE hospital, and DHT |
| Head of Computing | Data-entry at Farafenni field station, overseen by Maimuna Sowe |
| Transport Manager | Ongoing |
| Finance Manager | Ongoing |
| Personnel Manager | Ongoing |
| Administrative Director | Ongoing |
| Other services – specify | Modest requirement for printing forms to be done at Farafenni |

**Signature of principal investigator: Date:**

**__________________________________________________________________________**

**B Description of Project**

**B1 Background**

In response to the widespread occurrence of CQ-resistant parasites in Africa, and the more recent demonstrations that resistance to sulphadoxine/pyrimethamine (SP) is reducing the efficacy of that drug (1, 2,5), calls have been made for the deployment of new effective antimalarial combinations. Of particular interest are the artemisinins, which have short elimination half-lives, in various combinations with longer-acting drugs (11-14). Recent trials of AS in combination with SP in Africa have shown this to be a safe and efficacious regimen (9,11), but that a certain level of post-treatment transmission occurs (9). It is of importance to determine the effects of the artemisinins on *P. falciparum* transmission, and to use this information in the formulation of appropriate combination therapies for use in Africa.

We have run a series of trials in which we can measure *P. falciparum* gametocyte prevalence, density and infectivity to mosquitoes after treatment with artemisinins in various combinations with other anti-malarials. This work has been carried out at the MRC field station at Farafenni, The Gambia, during four consecutive malaria transmission seasons (1998 and 1999; ref. 7; 2000 and 2001, Sutherland et al., unpublished). In total 2,136 children have been treated with either CQ alone, CQ plus AS, SP alone, SP plus AS or SP plus CQ.

## Our studies in 1998/9 showed that AS, in combination with SP, reduced both the mean density of gametocytes in positive subjects, and the prevalence of gametocytes in the treated population (9) compared to SP alone. Nevertheless, a gametocyte prevalence of 5-10% was observed at day 7 in the combination group, and these gametocytes were capable of infecting mosquitoes in membrane-feeding experiments (9). Therefore, there may still be considerable transmission after treatment with AS combined with SP.

In 2000, we compared treatment with the combination CQ plus AS (3 days) with CQ alone. The results of this trial are now being prepared for publication, and can be summarised thus:

- CQ monotherapy exhibited a clinical failure rate of 13.3% and a cumulative parasitological failure rate of 75.6% over 28 days.
- The combination CQ/AS exhibited a clinical failure rate of 8.5% and a cumulative parasitological failure rate of 52.1% over 28 days.
- There was no significant difference between the two regimens in the total proportion of clinical failures over 28 days. The addition of AS to CQ prevented clinical failures occurring within 14 days of treatment, but provided no benefit in the latter half of follow-up.
- Gametocyte carriage was significantly reduced in the combination group up to day 14, but day 28 gametocyte carriage rates were high in both groups: 34% for CQ and 21% for CQ +AS.
- Children identified as gametocyte carriers on day 7, but who were gametocyte-negative on admission, were tested for infectiousness to *Anopheles*. Gametocyte-carriers from both groups were infectious, although gametocytes from children receiving CQ plus AS were less infective on average (Drakeley, Jawara and Sutherland, in preparation).

We conclude from this study that addition of AS to CQ does transiently reduce clinical failures and transmission immediately post-treatment. However, the cumulative parasitological failure rate of over 50%, and the high gametocyte carriage observed in the second fortnight of follow-up preclude this combination from any future role as first-line therapy in The Gambia.

Although none of the combinations so far tested are ideal for both transmission reduction and delivery of adequate clinical and parasitological efficacy, the best results have been obtained with SP / AS. Thus artemisinin combinations still hold the most promise in terms of both efficacy and public health benefit.

We propose a randomised controlled trial to compare the effect of Co-Artemether on post-treatment transmission. This will be compared to the effect of CQ combined with SP. CQ / SP provides a reference treatment group for comparison to our previous studies, as well as conforming to current Gambian Government guidelines for second-line treatment of uncomplicated malaria.

### B2 Project description

Project aim:

- Using a randomised control trial (RCT) design, measure the transmission potential of Gambian children after treatment with anti-malarial drugs.

Study aim for 2002:

- Measure *P.falciparum* transmission after Co-Artemether vs CQ plus SP by comparing gametocyte carriage rates and infectiousness to mosquitoes.

Secondary aims:

- To measure the prevalence of alleles associated with CQ drug resistance among pre-treatment isolates, parasitological treatment failures, emergent gametocytes and mosquitoes infected by membrane-feeding.

**Specific objectives:**

1. Using gametocyte prevalence, density and infectivity to mosquitoes by membrane-feeding as outcome measures, we plan to test whether Co-Artemether effectively reduces transmission compared to CQ / SP and, by reference to our previous work, compared to other antimalarial regimens.
2. Use PCR genotyping at loci implicated in CQ drug-resistance (8) and other polymorphic loci, in combination with data on treatment failures, to estimate the prevalence of resistant parasites in pre-treatment infections, in the emerging gametocyte population, and among treatment failures. Oocysts from infected mosquitoes will also be typed to look for evidence of drug selection in transmission. The relative contribution of resistant genotypes to circulating gametocyte pools will be estimated using the *in situ* PCR approach with our collaborators H. Babiker, L. Ranford-Cartwright and D. Walliker (3,4).
3. Use RT-PCR amplification of RNA from infected peripheral blood to establish stage specificity of parasite samples as recently described (7), and to examine genetic complexity of gametocyte populations *in vivo* by PCR analysis of polymorphic loci.

**Experimental Design and Methods to be Used**

###### Clinical trial and transmission experiments

Over the four previous seasons, we have established a randomised controlled trial (RCT) protocol, modified from the standard WHO guidelines for efficacy trials, for the examination of post-treatment transmission of falciparum malaria. In these trials based at the Farafenni Health Centre we recruited 600 children over 15 weeks, 500 children over 10 weeks, 536 children over 9 weeks and 500 children over 10 weeks respectively. These rates of subject recruitment, combined with accumulated gametocyte prevalence data will be fed into the design of the new protocol.

Children aged 1-10 years attending the Farafenni APRC Hospital or MCH clinic and requiring treatment for uncomplicated malaria will be recruited into the study after appropriate consent has been obtained from a parent or guardian. They will be randomly assigned to a treatment group by clinicians of the MRC Station, Farafenni, in a ratio that reflects *a priori* estimates of gametocyte carriage rates in the different treatment groups. Clinical and demographic data will be recorded on a standardised case report form (CRF) as in previous years. The principal investigator, slide readers and entomologists will be blinded as to the treatment group of each child. Field workers will be required to monitor drug doses on days 2 and 3 in the child’s home and therefore cannot be blinded. Blood films, blood spots and a microtainer blood sample will be obtained at the time of treatment. The blood sample will be used to measure PCV, genotyping parasites and previous antimalarial use.

On follow-up day 7, the child and a guardian will be brought to the field station for gametocyte-screening clinic, where duplicate thick films and filter-paper blood-spots will again be collected. Temperature and PCV will be measured and the mother and child questioned concerning any continuing clinical malaria symptoms. Each child will be asked to remain at the field station until one thick film has been read. If gametocytes are present, and permission given, approximately 3 ml of blood will be taken by venous puncture and immediately processed for mosquito infection studies.

All subjects will also be actively followed up at home on days 14 and 28 post-recruitment. On both occasions, 2 thick films will be taken, and blood spotted onto filter paper for genotyping. Parents and guardians will be encouraged to bring the child back to the clinic at any other time the child is unwell.

Farafenni MRC has two purpose-built insectaries. Previously, we have bred F1 generation *Anopheles gambiae* from wild caught mosquitoes for these experiments but Musa Jawara and colleagues have recently successfully established a stable colony of *A. gambiae* in the Farafenni insectaries which we will use as the source of adult mosquitoes for membrane-feeding in the coming season.

We will analyse the results of the trial for relevant clinical, parasitological and entomological parameters, with particular emphasis on transmission endpoints. Previously, we have used two summary statistics (the probability of post-treatment infectiousness, and the infectious proportion of subjects) that incorporate gametocyte prevalence, density and infectivity to mosquitoes (9). These data will then be related to the estimated proportion of treated infections in rural areas of The Gambia to derive an estimate of the contribution that children treated with each regimen would make to transmission.

###### Parasite genotyping

Along with our collaborators at Edinburgh University, we are now establishing the necessary capacity to perform basic molecular biology in the MRC laboratories at Farafenni. The two collaborating groups have provided a PCR machine and electrophoresis equipment from the Gates Malaria Programme at LSHTM, and the MRC project funding held by Dr Babiker and Prof. Walliker in Edinburgh. Our collaborators’ MRC funding is also supporting a higher scientific officer with experience in molecular biology (Dr. Davis Chibuzo Nwakanma), and our Gates Malaria Partnership funding will allow us to provide any further training he requires in the specific area of drug-resistance genotyping which is to be used in these and other studies.

A novel application of RT-PCR has allowed us to detect expression of developmental stage-specific genes in peripheral blood obtained at the time of feeding mosquitoes (7). RT-PCR of an asexual-specific locus (*resa*) and a gametocyte-specific locus (*pfs16*) permits samples with gametocytes only and samples with sub-patent asexual parasites as well to be distinguished from each other. Thus we can clarify whether the DNA in our genotyping experiments is derived just from gametocytes, or from a combination of gametocytes and asexual parasites, within the limits of detection of our RT-PCR system. The results of this molecular genetics approach have clearly demonstrated that day 4 and day 7 circulating gametocytes comprise a mix of, on average, two or more genotypes. Thus in our study area the requirements are being met for heterologous genetic recombination to occur in the mosquito. This is the first time such an analysis has been reported. However, this work included only 10% of our study subjects, and a meaningful comparison among treatment groups was not possible with this sample size. We plan an expansion of this work to other drug treatment groups, and with larger sample sizes, using these methodologies.

We propose to determine the genotype of selected parasite samples at loci involved in drug resistance. We will type at both the *pfmdr1* and *pfcrt* loci implicated in resistance to CQ. These analyses will provide crucial information as to the involvement of these markers in any treatment failures among SP / CQ-treated children, with the Co-Artemether group as comparitor. We would predict that this group would not show evidence of selection for CQ-resistance. We will also be able to determine the relative contribution of drug-resistant loci to the emerging gametocyte pool as in previous studies with CQ-resistance (8). Further, using the combination of chemotherapy trials with mosquito infectivity studies, we will be able to follow drug-resistant genotypes into the mosquito, by dissecting oocysts from infected mosquitoes and typing them with established PCR methods (4,8). The technique of *in situ* PCR, to be performed in collaboration with our university of Edinburgh colleagues and Dr Lisa Ranford-Cartwright in Glasgow, will enable us to evaluate the relative abundance of gametocytes carrying drug-resistance loci in our gametocyte samples prior to feeding to mosquitoes. The subsequent typing of single oocysts from mosquitoes successfully infected with those same gametocytes will then allow us to evaluate the post-transmission abundance of these same loci.

1. Kublin JG, Dzinjalamala FK, Kamwendo DD, Malkin EM, Cortese JF, Martino LM, Mukadam RA, Rogerson SJ, Lescano AG, Molyneux ME, Winstanley PA, Chimpeni P, Taylor TE, Plowe CV. 2002. Molecular markers for failure of sulfadoxine-pyrimethamine and chlorproguanil-dapsone treatment of Plasmodium falciparum malaria. *J Infect Dis.* **185:** 380-388.
2. Mutabingwa T, Nzila A, Mberu E, Nduati E, Winstanley P, Hills E, Watkins, W. 2001. Chlorproguanil-dapsone for treatment of drug-resistant falciparum malaria in Tanzania. *Lancet* **358:** 1218-1223.
3. Ranford-Cartwright, L.C., and Walliker, D. 1999. Intragenic recombinants of *Plasmodium falciparum* identified by in situ polymerase chain reaction *Mol. Biochem. Parasitol.* **102:** 13-20.
4. Ranford-Cartwright, L.C., Balfe, P., Carter, R. and Walliker, D. 1991. Genetic hybrids of *Plasmodium falciparum* identified by amplification of genomic DNA from single oocysts. *Mol. Biochem. Parasitol.* **49:** 239-244.
5. Ronn, A.M., Msangeni, H.A., Mhina J., Wernsdorfer W.H., Bygbjerg I.C. 1996. High level of resistance of *Plasmodium falciparum* to sulfadoxine-pyrimethamine in children in Tanzania. *Trans R Soc Trop Med Hyg*. **90:** 179-81.
6. Standard drug treatment guidelines. 1998 Dept for Health and Women’s Affairs, The Gambia.
7. Sutherland C, Alloueche A, McRobert L, Ord R, Leggat J, Snounou G, Pinder M, Targett G, 2002. Genetic complexity of *P. falciparum* gametocytes isolated from the peripheral blood of treated Gambian children. *Am J Trop Med Hyg* (in press).
8. Sutherland CJ, Alloueche A, Curtis J, Drakeley CJ, Ord R, Duraisingh M , Greenwood, BM, Pinder M, Warhurst, DC, Targett GAT. 2002b. Gambian children successfully treated with chloroquine can harbour and transmit *Plasmodium falciparum* gametocytes carrying resistance genes. *Am J Trop Med Hyg* (submitted).
9. Targett, G.A.T., Drakeley, C.J., Jawara, M., von Seidlein, L., Coleman, R., Deen, J., Pinder, M., Doherty, T., Sutherland, C., Walraven, G., and Milligan, P. 2000. The effects on transmission of *Plasmodium falciparum* malaria of treatment with pyrimethamine/ sulphadoxine alone or in combination with artesunate. 2001; 183: 1254-1259
10. Trigg JK, Mbwana H, Chambo O, Hills E, Watkins W, Curtis CF. 1997. Resistance to pyrimethamine/sulfadoxine in *Plasmodium falciparum* in 12 villages in north east Tanzania and a test of chlorproguanil/dapsone. *Acta Trop* **63:** 185-189.
11. von Seidlein L, Milligan P, Pinder M, Bojang K, Anyalebechi C, Gosling R, Coleman R, Ude JI, Sadiq A, Duraisingh M, Warhurst D, Alloueche A, Targett G, McAdam K, Greenwood B, Walraven G, Olliaro P, Doherty T. 2000. Efficacy of artesunate plus pyrimethamine-sulphadoxine for uncomplicated malaria in Gambian children: a double-blind, randomised, controlled trial.*Lancet*. **355:** 352-7.
12. von Seidlein, L., Bojang, K., Jones, P., Jaffar, S., Pinder, M., Obaro, S., Doherty, T., Haywood, M., Snounou, G., Gemperli, B., Gathmann, I., Royce, C., McAdam, K., and Greenwood, B. 1998. A randomized controlled trial of artemether/benflumetol, a new antimalarial and pyrimethamine/sulfadoxine in the treatment of uncomplicated falciparum malaria in African children *Am. J. Trop. Med. Hyg.* **58:** 638-644.
13. Von Seidlein, L., Jaffar, S., Pinder, M., Hatwood, M., Snounou, G., Gemperli, B., Gathmann, I., Royce, C., and Greenwood, B. 1997. Treatment of African children with uincomplicated falciparum malaria with a new antimalarial drug, CGP 56697. *J. Infect. Dis.* **176:** 1113-6.
14. White NJ, Nosten F, Looareesuwan S, Watkins WM, Marsh K, Snow RW, Kokwaro G, Ouma J, Hien TT, Molyneux ME, Taylor TE, Newbold CI, Ruebush TK 2nd, Danis M, Greenwood BM, Anderson RM, Olliaro P. 1999. Averting a malaria disaster. *Lancet* **353:** 1965-1967

**B3 Details of study design and investigations**

1. **What type of study design is proposed (eg case control, prospective cohort, randomised controlled trial, descriptive etc)**

Randomised Controlled Trial

1. **What is the proposed size of the study (this may relate to patients, cases, controls, survey subjects, laboratory samples etc, as appropriate).**

The aim is to recruit approximately 550 patients over 11 weeks.

1. **Please describe the statistical considerations and sample size calculations involved in determining the size of the study.** (If you do not have access to statistical advice, please consult the MRC Laboratories Statistics Department.)

Approximately 550 patients will be treated.

In our previous studies the lowest observed gametocyte rate 7 days after treatment was ~8% (excluding those who had gametocytes on day 0), seen after treatment with CQ + artesunate. We expect that the rate after Co-Artemether will also be 5-10%. Gametocyte rates after CQ / SP treatment are much higher, between 40% and 50% at day 7. We will aim to enrol 450 subjects in the Co-Artemether group, and 100 subjects in the group receiving CQ / SP. All treated children will be screened for gametocytes on day 7 only. We expect to be able to perform 20 to 40 membrane feeds and 40 to 50 membrane feeds respectively in the Co-Artemether and CQ / SP treatment groups. This will provide a power of 80% at the 95% confidence level to detect a 9-fold difference in average probability of mosquito infection (as observed between SP / AS and SP in 1999). We will have a power of 80% at the 90% confidence level to detect a five-fold difference in the proportion of infectious children in the two groups (as observed in 1999).

**For studies involving human subjects:**

1. **How and where will the study subjects (cases, controls, etc) be selected? Has it been confirmed that they are not already involved in other studies?**

Subjects will be enrolled at Farafenni AFPRC Hospital/MCH clinic from among children presenting with uncomplicated falciparum malaria. Recruits will be randomised among the treatment groups. No subjects involved in any concurrent intervention studies in the Farafenni area will be enrolled in this study. However, children who are part of passive surveillance studies currently underway in the Farafenni area may participate in our study, and close co-operation has been agreed with the PI of the surveillance studies, Dr K. Bojang.

1. **What inclusion/exclusion criteria will be applied?**

##### Children aged 1-10 years and the informed consent from a parent or guardian.

##### Inclusion Criteria

Fever or history of fever and a parasitaemia > 500/ul of *P.falciparum*

Informed consent from a parent or guardian.

Aged 1-10 years.

##### Exclusion Criteria

Signs or symptoms of severe malaria including severe anaemia (PCV <20% or Hb<5g/dL), hyperparasitaemia (>500 parasites per hpf, ie 250000 per ul) or convulsions.

**Children displaying any of the above signs will be directly admitted to the paediatric ward of the APRC Hospital** **by the recruiting physician (SD, GW).**

Vomiting or inability to take drugs orally. **Alternative treatment will be administered.**

A history of anti-malarial treatment in the past two weeks.

Evidence of any other chronic or acute illness.

Infection or co-infection with non-falciparum malaria parasites.

Patent gametocytaemia.

1. **How will informed consent be obtained?**

Informed consent will be obtained from a parent or guardian at the time of enrolment into the study (Consent form attached). The aims and methods, including details of blood samples needed, will be explained in a language they understand. The recruiting field worker/nurse will sign that consent was given. The right of all individuals not to participate or to withdraw their consent will be respected and the children will then receive standard treatment. Parents may withdraw their consent at any point in the trial and the child’s treatment will not be affected.

1. **What samples, if any, will be taken and what investigations will be conducted?**

- Finger-prick blood samples on days 0, 7, 14 and 28 to provide thick films for parasite detection and filter paper blood spots for molecular typing of parasites.
- Microtainer samples on day 0 for PCV, genotyping and RT-PCR
- 2-3ml blood by venepuncture on day 7 from consenting gametocyte-positive patients for membrane feeding of mosquitoes.
- Alcohol-fixed oocysts from infected mosquito guts for genotyping

1. **Will treatment be given? YES**

**If yes:**

**Nature of treatment(s)**

For drugs: dosage and duration of treatment

- Co-Artemether will be given as recommended: 6 paediatric doses in units of 10mg artemether/60mg benflumetol. 1, 2, 3 or 4 such units will be administered to children in the weight classes 5-10kg, 10-15kg, 15-20kg and 20-25kg respectively. The 6 doses will be supervised at recruitment (ie 1000 to 1230 hr) and between 1800 and 2000hr on day zero, and between 0700 and 0930hr and between 1800 and 2000hr on days 1 and 2.
- CQ / SP will be administered as a single dose of SP [for children ≤ 10kg this comprises 0.5 tablet Fansidar® (12.5mg sulphadoxine and 250mg pyrimethamine), with an additional quarter tablet for each additional 5kg] PLUS three consecutive daily doses of CQ, giving a total dose of 25mg/kg chloroquine over 3 days.
- Each child will also be given a stat. dose of paracetamol (10 mg/kg) at enrolment.

Children will be observed for 30 minutes for vomiting. Any child who vomits the study medication within this time will receive a second dose. Children who vomit a second time will not be recruited and treated with sub-cutaneous CQ.

Person(s) responsible for administering treatment

- Farafenni malaria epidemiologist (SD) and other MRC clinicians (GW)

1. **For questionnaires/interviews, who will be conducting these?**

- Senior Field Assistant (Yorro Bah) and nurses seconded from the hospital and MCH.

1. **Who will be primarily responsible for the statistical design and analysis?**

- The principle investigator and PM, in collaboration with LSHTM and MRC statisticians.

1. **Who will be primarily responsible for data management?**

- The Farafenni data manager in collaboration with the principle investigator.

**C Ethical issues**

**Please highlight any potential ethical issues and how you propose to deal with these. What outcomes and benefits will derive from the study? How will the results of the study contribute to the health of the people of the Gambia?**

This section, together with B3 is particularly important to the Ethical Committee and should be comprehensive. Please continue on a separate sheet if necessary.

This is a continuation of studies designed to establish the drug or drugs most appropriate for use in interrupting transmission of malaria to mosquitoes and to demonstrate the benefits, if any, gained by use of associating artemisinins with other anti-malarials. The primary benefit to the participants is that, as well as receiving free treatment, after treatment at the clinic they will be monitored by field assistants visiting their homes. In addition, the patients have access to study clinicians at the daily recruiting clinic run by the research team in the old Farafenni Health Centre, or at MRC Farafenni Field Station should the need arise.

The benefit of selecting a drug(s) that prevent transmission comes with its use for mass treatment of a population prior to the transmission season. The expectation is that this would significantly reduce the source of malaria infection in the subsequent malaria season, with a consequent reduction in malaria morbidity and mortality.

The major ethical issue of the current proposal is treatment safety. Co-Artemether has previously been the subject of safety and efficacy trials in The Gambia and was proved safe and efficacious (12, 13). The combination CQ / SP is recognised as the second-line treatment of choice for uncomplicated malaria in The Gambia and is widely used in this role.

Collection of blood samples beyond the initial fingerprick required for diagnosis in the recruitment clinic will be done only after informed consent has been obtained.

Any child in the study in need of additional medical attention will be provided with the appropriate treatment or referred.

Any child who fails treatment and returns with symptoms of clinical malaria will be given rescue medication of oral quinine.

**Appendix 1 – Consent Form.**

## MRC Farafenni Targett 2002/ G02 Consent Form

**Patient’s Name:________________________ Study Number:____________**

The following should be explained to the mother or guardian of the participant in the language s/he understands.

Your child has malaria, a common and serious disease in Gambian children. There are many drugs that can be used to treat malaria. In the Gambia, chloroquine is usually used to treat children with mild malaria and Fansidar is used in some cases. These drugs still work but not as well as they used to do. New drugs are needed. Recently a new drug, Co-Artemether, has been tested and seems to work well in patients with malaria and in those who cannot be treated with chloroquine or Fansidar.

Most of the time, a few days after taking chloroquine or Fansidar, the patient feels better and the malaria parasite gradually disappears from their blood. But during this time, if mosquitoes bite the patient then the mosquitoes can become infected with the malaria parasite and give the disease to the next person they bite. We want to find out if after treatment with the new drug Co-Artemether, fewer patients can pass the malaria parasite onto mosquitoes than patients treated with Fansidar and chloroquine. Co-Artemether was found to be safe for children in a trial in The Gambia.

If you agree that your child can enter this trial, then your child will receive Fansidar and chloroquine together or Co-Artemether. We will also give you paracetamol to take today. One of our field assistants will visit you over the next 2 days to ask you if the child is well and give the medicines. We will bring you and your child to the MRC compound in 7 days’ time. If your child has parasites that may infect mosquitoes we would like to draw a teaspoon (3ml) of blood from your child’s arm.

To make sure your child is well and no longer has parasites we will come to your house in 14 days and 28 days, and examine and finger prick your child.

MRC, The Gambia and the malaria research group from the London School of Hygiene and Tropical Medicine in UK are doing this research.

All the information you give to us will be treated as confidential. If you have any questions or queries during the study, Dr Sam Dunyo, MRC Farafenni will be happy to talk to you about them.

- Your child does not have to join this study. If s/he joins, s/he can leave whenever you wish and s/he will receive the usual treatment.
- Do you agree that your child can enter this trial?

**Name of parent/guardian:………………………………………**

I confirm that I have explained to the parent or the guardian of the above mentioned child in an appropriate language, that s/he understood what had been said and that s/he agreed freely that their child could enter this trial.

**Name of the interpreter:…………………………Signature:…………………Date:………………….**

### Name of the investigator:………………………. Signature:…………………Date:…………………
